# Factors associated with overdiagnosis of benign pulmonary nodules as malignancy: a retrospective cohort study

**DOI:** 10.1186/s12890-023-02727-7

**Published:** 2023-11-21

**Authors:** Xirui Duan, Zhiqiang Ouyang, Shasha Bao, Lu Yang, Ailin Deng, Guangrong Zheng, Yu Zhu, Guochen Li, Jixiang Chu, Chengde Liao

**Affiliations:** 1 Department of Radiology, Yan’an Hospital of Kunming City (Yan’an Hospital Affiliated to Kunming Medical University; Yunnan Cardiovascular Hospital), Kunming, China; 2 Department of Radiology, Yunnan Cancer Hospital/Center, Third Affiliated Hospital of Kunming Medical University, Kunming, China

**Keywords:** Benign pulmonary nodules, Overdiagnosis, Preoperative model, Imaging features, Malignant pulmonary nodules

## Abstract

**Objective:**

To establish a preoperative model for the differential diagnosis of benign and malignant pulmonary nodules (PNs), and to evaluate the related factors of overdiagnosis of benign PNs at the time of imaging assessments.

**Materials and methods:**

In this retrospective study, 357 patients (median age, 52 years; interquartile range, 46–59 years) with 407 PNs were included, who underwent surgical histopathologic evaluation between January 2020 and December 2020. Patients were divided into a training set (*n* = 285) and a validation set (*n* = 122) to develop a preoperative model to identify benign PNs. CT scan features were reviewed by two chest radiologists, and imaging findings were categorized. The overdiagnosis rate of benign PNs was calculated, and bivariate and multivariable logistic regression analyses were used to evaluate factors associated with benign PNs that were over-diagnosed as malignant PNs.

**Results:**

The preoperative model identified features such as the absence of part-solid and non-solid nodules, absence of spiculation, absence of vascular convergence, larger lesion size, and CYFRA21-1 positivity as features for identifying benign PNs on imaging, with a high area under the receiver operating characteristic curve of 0.88 in the validation set. The overdiagnosis rate of benign PNs was found to be 50%. Independent risk factors for overdiagnosis included diagnosis as non-solid nodules, pleural retraction, vascular convergence, and larger lesion size at imaging.

**Conclusion:**

We developed a preoperative model for identifying benign and malignant PNs and evaluating factors that led to the overdiagnosis of benign PNs. This preoperative model and result may help clinicians and imaging physicians reduce unnecessary surgery.

## Introduction

Lung cancer remains one of the most prominent causes of cancer-related deaths worldwide, with its incidence and mortality rates demonstrating a marked increase in recent years. Between 2000 and 2016, there was a 0.8% annual increase in the age-standardized incidence, while new cases and deaths surged by 162.6% and 123.6%, respectively [1]. Early-stage lung cancer has no obvious clinical symptoms but is detected by computed tomography (CT) as pulmonary nodules (PNs). Annual screening with LDCT is recommended for high-risk individuals by guidelines from the U.S. Preventive Services Task Force (USPSTF),and this recommendation increases the demand for low-dose CT (LDCT) for the patient [2]. With the development of CT technology and the proliferation of lung cancer screening programs, the detection rate of PNs is increasing, which may cause public anxiety [3]. However, despite the identification of numerous clinical-pathologic factors (e.g., age, sex, smoking history, family history of cancer, lesion type, lesion size, lesion location, biomarker, and image feature) that have been associated with the nature of PNs, the definitive predictive factors that distinguish between benign and malignant PNs remain elusive [4, 5]. The relative importance and interrelationship of these factors are unclear, and with current knowledge, it is difficult to accurately predict which PNs are at risk for over-diagnosis.

To discriminate between the benign and malignant PNs, lung cancer screening programs have been implemented in many countries. However, there are some controversies and risks associated with lung cancer screening. One of the risks is false-positive results. A meta-analysis revealed that screening leads to a higher long-term cumulative incidence of lung cancer (1.51; 95% CI: 1.06–2.14), with an estimated 49% of screen-detected cancers potentially being over-diagnosed [6]. A study of the National Lung Screening Trial were drew a similar result as well [7]. In a screening program, false-positive results are associated with increased healthcare costs, patient anxiety, and morbidity or mortality related to diagnosis and treatment [8]. In an analysis of over 9000 lung cancer screening examinations, the frequencies of malignancy in Lung-RADS 4A, 4B and 4X nodules were 15.5%, 36.3%, and 76.8%. Therefore, the majority of suspicious nodules that undergo additional work-up, and intervention were in fact benign [9]. Many patients experienced unnecessary surgery or biopsy due to the false-positive results, in which the mainstream choice for PN is minimally invasive video-assisted thoracoscopic surgery (VATS) [10]. However, because the small nodules are difficult to locate with the tactile sensation or the naked eye, wedge resection under VATS for ground-glass opacity nodules (GGN) is challenging. The frequency of complications is estimated to be 3–4% of treated patients, of which prolonged postoperative air leak is the most frequent and the other significant complications are bleeding, infections, postoperative pain, and recurrence at the port site [11]. These inevitably increase the risk and pain of patients. What’s more, observations of a review found that at least 95% of PNs are benign, which are most commonly granulomas or intrapulmonary lymph nodes [12].

The objective of this study is to analyze the imaging features of nodules in preoperative chest CT scans of patients who have undergone surgery or biopsy, and to establish a preoperative prediction model for nodules that incorporates the patients' clinical and pathological features. This model aims to minimize the rate of unnecessary surgery for benign PNs. Moreover, the study seeks to pinpoint specific factors that contribute to the classification of benign PNs.

## Materials and methods

### Patient selection

**Fig. 1:**
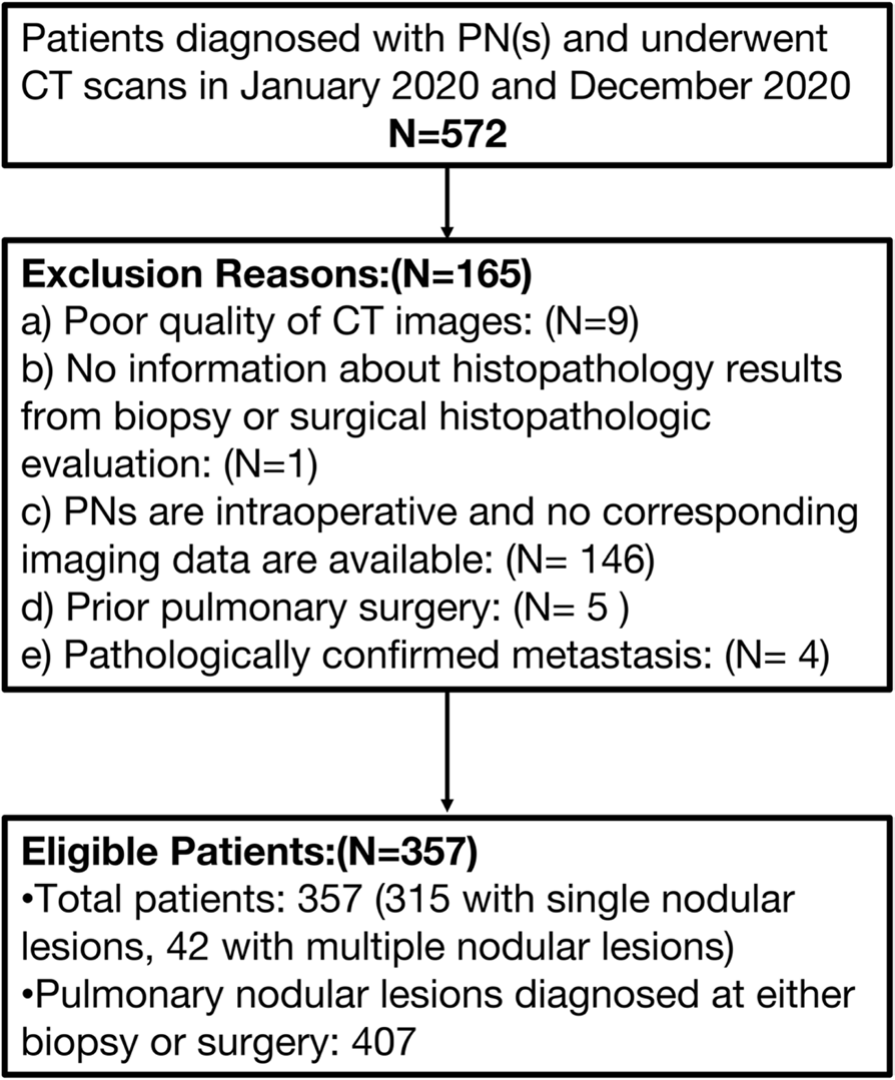
Patient Inclusion and Exclusion Criteria Flow Diagram

This study was approved by the institutional review board, and the requirement for informed consent was waived due to its retrospective design. Patients who were diagnosed with PN(s) and underwent spiral CT scans at our institution between January 2020 and December 2020 were initially considered eligible for our study (Fig. 1). The inclusion criteria were as follows: (a) age ≥ 18 years old;(b) nodule size of 5 mm to 15 mm;(c) diagnosis of PN(s) by postoperative pathologic examination;(d) clear pathologic results and (e) no history of cancer. Patients were excluded due to the following reasons:(a) poor quality of CT images, (b) no information about histopathology results from biopsy or surgical histopathologic evaluation, (c) PN(s) were intraoperative and no corresponding imaging data are available, (d) prior pulmonary surgery, and (e) pathologically confirmed metastasis.

All data was extracted from the database of our hospital. The generic perioperative information of patients was reviewed including demographic information (age, sex, smoking history, family history of cancer, and biomarker positive), pathological information, and surgical details (approach of operation, extent of resection, and resection location).

### Images acquisition

All the patients included in the study underwent non-contrast CT (NCCT) as imaging data before lung nodule resection or biopsy, which was an interval of fewer than 14 days from NCCT. The chest CT was performed with Siemens (SOMATOM Force), Canon (Aquilion PRIME), and Philips (Ingenuity CT) scanners using the following same acquisition parameters: layer thickness, 1.0 mm; tube voltage, 120 kvp; tube current-exposure time product, 160 mA. All images were set with a standard lung window (window width 1600HU; window position, -600HU) window. Images were transferred to the Picture Archiving and Communication System (PACS) system.

### Images analysis

Two chest radiologists (Bao Shasha, a third-year post-graduate student and Deng Ailin, a third-year post-graduate student) were double-blinded to review the lesion and surrounding structures according to the American College of Radiology Lung-Reporting and Data System and to identify the possibility of PNs being benign or malignant. According to this system, Lung-RADS 1 or 2 lesions are generally considered benign due to their low risk of malignancy, while Lung-RADS 4B or 4X lesions are classified as malignant given their high malignancy risk. The malignancy status of Lung-RADS 3 or 4A lesions largely depends on the expertise of radiologists in differentiating benign from malignant PNs. When there is a disagreement, it is defined by group discussion between the two mentioned above and Xirui Duan (a first-year post-graduate student). In previous studies and models [13–17], spiculation, pleural retraction, vascular convergence and air bubble sign have also been used as one of the imaging risk factors for radiologists to distinguish between benign and malignant PNs. Recent review also supports these imaging features in evaluating PNs [12]. Therefore, the nodular lesions were retrospectively categorized according to imaging findings as follows: (a) spiculation: a radial and unbranched striated shadow extending from the boundary of the PNs to the surrounding parenchyma of the pulmonary; (b) pleural retraction: a retraction of adjacent pleura toward the nodule; (c) vascular convergence: vessels are clustered internally or abnormally inclined toward the nodules compared with the normal pulmonary parenchyma; or (d) air bubble sign (vacuolar sign): a small air-containing space < 5 mm in length within the PNs.

### Dataset allocation

All pulmonary nodular lesions were classified into one of two groups based on the final histopathology results; the final histopathology result was defined as benign or malignant by surgical or biopsy histopathologic evaluation. Overdiagnosis was defined when a benign PN at biopsy or surgery was assessed as high risk of malignancy on CT scan. The included lesions were randomly assigned to the training and validation data set at a ratio of 7:3.

### Logistic regression analysis

Clinical, histopathologic, and imaging features were also evaluated for all benign pulmonary nodular lesions at biopsy or surgery to investigate the factors associated with benign PNs over-diagnosed at CT scan by logistic regression analysis. Specifically, an bivariate logistic regression analysis was performed on the training set to identify factors associated with a benign PN. A multivariable logistic regression analysis was conducted by using variables selected according to their clinical meaning and statistical significance (*p*, 0.05). The bivariate and multivariable logistic regression mentioned above will be used to identify factors associated with the over-diagnosis of benign PNs too. Multiple imputations were applied for the missing values, which used a fully conditional specification method; pooled adjusted ORs with 95% CIs were provided after 5 multiple imputations [18]. The predictive performance of the training set was calculated as the median value of the 5 results for the missing imputations. However, we obtained the receiver operating characteristic curve through complete case analysis.

### Model validation

The developed multivariable regression model was then validated with the validation set. The model's discrimination capability was evaluated using the area under the receiver operating characteristic curve (ROC), which is equivalent to the Harrell's c-statistic for binary results. The goodness of fit was assessed by using the Hosmer–Lemeshow test. The association between the observed and predicted probabilities of a benign PN was visually displayed through a calibration plot. The receiver operating characteristic curve and calibration plot for the validation set were derived from a complete case analysis, without employing multiple imputations. In real-world clinical situations, where minimizing biopsies is crucial to reduce overdiagnosis, a predictive model developed without relying on biopsy outcomes or histopathological data may be more appropriate.

### Analysis of over-diagnosed benign PNs

Clinical, histopathologic, and imaging features were also evaluated for all benign pulmonary nodular lesions at biopsy or surgery to investigate the factors associated with benign PNs over-diagnosed at CT scan by multivariable logistic regression analysis.

### Statistical analysis

Statistical analysis was performed with software (SPSS version 27.0.1, SPSS for Statistical Computing; and GraphPad Prism version 8.0.2, The ROC curve and Calibration plot were established by GraphPad Prism). Continuous variables are expressed as medians and interquartile ranges depending on their distribution and were compared by using the Mann–Whitney U test. Categorical variables are expressed as numbers with percentages and were compared by using the x^2^ test or Fisher exact test. The results were considered statistically significant with two-tailed analyses, with *p* values less than 0.05.

## Results

### Clinical and pathological characteristics

A total of 357 patients were included in the study, consisting of 129 males (36.1%) and 228 females (63.9%). The median age of the 357 patients with 407 pulmonary nodular lesions was 52 years (interquartile range, 46–59 years). Among the 407 pulmonary nodular lesions, 163 lesions (40%) were diagnosed using thoracoscopic wedge resection, 156 lesions (38.3%) were diagnosed using thoracoscopic lobectomy, 86 lesions (21.1%) were diagnosed using thoracoscopic partial lobectomy, and 2 lesions (0.5%) were diagnosed using needle biopsy of the lung. In the final histopathologic evaluation, 160 of the 407 pulmonary nodular lesions (39.3%) were determined to be benign.

Out of the 357 patients, we allocated 285 patients to the training set and the remaining 122 patients to the validation set. The patient demographic characteristics, along with the baseline clinical, imaging, and pathologic characteristics of the training set (*n* = 285) and validation set (*n* = 122), are presented in Table 1.

**Table 1:** Clinical and pathologic characteristics of patients and lesions in training and validation set

	Training Set			Validation Set		
Variable	Malignant nodules	Benign nodules	*P* Value	Malignant nodules	Benign nodules	*P* Value
No.of lesions	188	97		85	37	
Median age (y)	53(46–59)	53(46–59)	.929	51(47–58)	51(46–55)	.690
Sex			.531			.624
Male	59(31.4)	34(35.1)		35(41.2)	17(45.9)	
Female	129(68.6)	63(64.9)		50(58.8)	20(54.1)	
Smoking history			.617			.039
No	152(80.9)	76(78.4)		66(77.6)	22(59.5)	
Yes	36(19.1)	21(21.6)		19(22.4)	15(40.5)	
Family history of cancer			.408			.126
No	160(85.1)	86(88.7)		72(84.7)	35(94.6)	
Yes	28(14.9)	11(11.3)		13(15.3)	2(5.4)	
Biomarker positive^a^						
CEA positive(%)*n* = 300	24.3(34/140)	13.4(9/67)	.072	18.5(12/65)	17.9(5/28)	.945
NSE positive(%)*n* = 271	9.2(12/130)	11.5(7/61)	.629	3.4(2/58)	9.1(2/22)	.301
CYFRA21-1 positive(%)*n* = 297	25.4(35/138)	40.9(27/66)	.024	24.6(16/65)	28.6(8/28)	.689
SCC positive(%)*n* = 300	5.7(8/140)	4.5(3/67)	.711	6.2(4/65)	3.6(1/28)	.613

Unless otherwise noted, variables are expressed as numbers of patients with percentages in parentheses, or as medians, with interquartile ranges in parentheses. *CEA* Carcinoma Embryonic Antigen, *NSE* Neuron Specific Enolase, *CYFRA21-1* Cytokeratin 19 fragment, *SCC* Squamous Cell Carcinoma Antigen

^a^Data in parentheses are numerator/denominator; the number of patients for whom histopathological data were available for each molecular marker is given

### Imaging characteristics

Compared with malignant PNs, benign PNs more frequently were solid nodules (79.4% vs. 26.1% in the training set; 86.5% vs. 21.2% in the validation set; *P* < 0.001). Conversely, malignant PNs more frequently were non-solid nodules (46.3% vs. 12.4% in the training set; 43.5% vs. 13.5% in the validation set; *P* < 0.001) and part-solid nodules(27.2% vs. 8.2% in the training set; 35.3% vs. 0% in the validation set; *P* < 0.001); malignant PNs were potentially more frequently manifested vascular convergence (78.7% vs. 56.7% in the training set, *P* < 0.001; 76.5% vs. 59.5% in the validation set, *P* = 0.056). Unfortunately, at our institution median lesion size, location, spiculation, pleural retraction and vacuolar sign were not statistically significant in identifying benign and malignant PNs (Table 2).

**Table 2:** Imaging characteristics of patients and lesions in training and validation set

	Training Set			Validation Set		
Variable	Malignant nodules	Benign nodules	*P* Value	Malignant nodules	Benign nodules	*P* Value
Lesion type			< .001			< .001
Solid nodules	49(26.1)	77(79.4)		18(21.2)	32(86.5)	
Part-solid nodules	52(27.7)	8(8.2)		30(35.3)	0(0)	
Non-solid nodules	87(46.3)	12(12.4)		37(43.5)	5(13.5)	
Median lesion size at imaging (mm)	8.5(6.5–11.5)	7.5(6–10)	.033	8.5(7–11)	9.5(7–11.5)	.554
Location			.347			.150
Left upper lobe	43(22.9)	19(19.6)		26(30.6)	6(16.2)	
Left lower lobe	27(14.4)	22(22.7)		8(9.4)	5(13.5)	
Right upper lobe	64(34.0)	24(24.7)		32(37.6)	14(37.8)	
Right middle lobe	17(9.0)	8(8.2)		9(10.6)	2(5.4)	
Right lower lobe	36(19.1)	23(23.7)		10(11.8)	9(24.3)	
Subpleural	1(0.5)	1(1.0)		0(0)	1(2.7)	
Imaging finding						
Spiculation			.015			.077
No	65(34.6)	48(49.5)		25(29.4)	17(45.9)	
Yes	123(65.4)	49(50.5)		60(70.6)	20(54.1)	
Pleural retraction			.683			.581
No	119(63.3)	59(60.8)		53(62.4)	25(67.6)	
Yes	69(36.7)	38(39.2)		32(37.6)	12(32.4)	
Vascular convergence			< .001			.056
No	40(21.3)	42(43.3)		20(23.5)	15(40.5)	
Yes	148(78.7)	55(56.7)		65(76.5)	22(59.5)	
Vacuolar sign			.008			.697
No	101(53.7)	68(70.1)		45(52.9)	21(56.8)	
Yes	87(46.3)	29(29.9)		40(47.1)	16(43.2)	

Unless otherwise noted, variables are expressed as numbers of patients with percentages in parentheses, or as medians, with interquartile ranges in parentheses

### Bivariate logistic regression analysis of factors associated with benign PNs

**Fig. 2:**
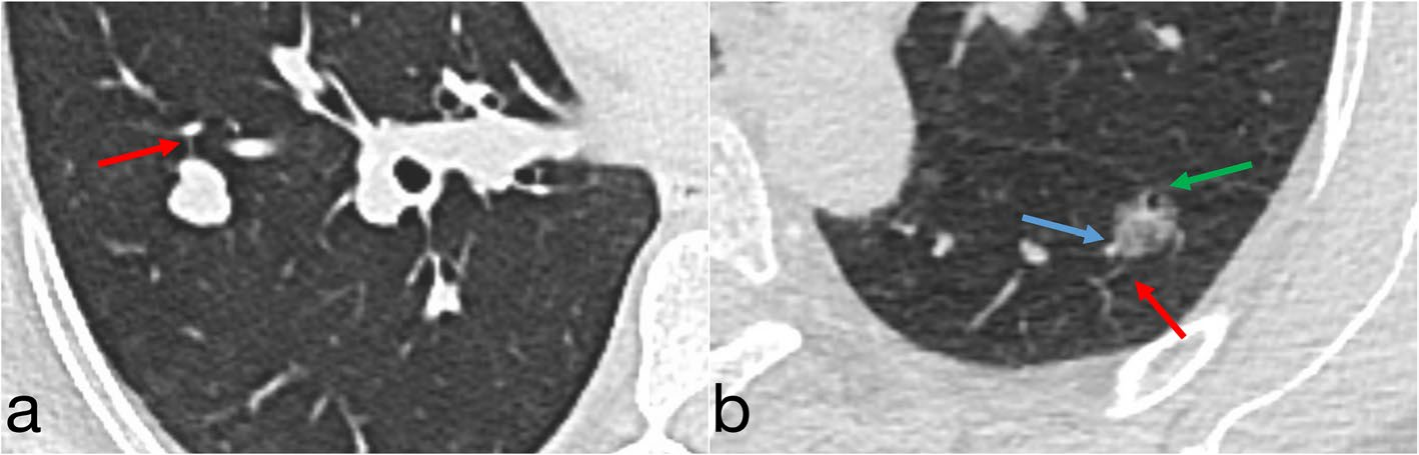
Computed tomography image shows vascular convergence (blue arrows), pleural retraction (green arrows), and spiculation (red arrows). **a** a solid nodule with an average diameter of 1.1 cm in the right lower lobe of a 54-year-old woman’s lung and pathologically confirmed to be benign PN; **b** a part-solid nodule with an average diameter of 1.1 cm in left lower lobe of 58-year-old woman’s lung and pathologically confirmed to be malignant PN

At bivariate logistic regression analysis, part-solid nodules (OR, 0.98; 95% CI: 0.04, 0.22; *P* < 0.001), non-solid nodules (OR, 0.88; 95% CI: 0.04, 0.18; *P* < 0.001), lesion size at imaging (OR, 0.91; 95% CI: 0.83, 1.00; *P* = 0.041),spiculation (OR, 0.54; 95% CI: 0.33, 0.89; *P* = 0.015), vascular convergence (OR, 0.35; 95% CI: 0.21, 0.60; *P* < 0.001), and vacuolar sign (OR, 0.50; 95% CI: 0.29, 0.83; *P* = 0.008)at histopathology were inversely associated with benign PNs.CYFRA21-1(OR, 2.01; 95% CI: 1.07, 3.76; *P* = 0.029)were positively associated with benign PNs (Table 3, Fig. 2). Smoking history, family history of cancer, lesion size at imaging, image findings of Pleural retraction and vacuolar sign, location, and biomarker of CEA, NSE, and SCC were not associated with benign PNs.

**Table 3:** Bivariate logistic regression analysis to predict benign nodules in training set

	Bivariate Analysis	
Variable	Odds Ratio	*P* Value
Smoking history		
No	1(reference)	
Yes	1.17(0.64–2.14)	.617
Family history of cancer		
No	1(reference)	
Yes	0.73(0.35–1.54)	.410
Lesion type		
Solid nodules	1(reference)	
Part-solid nodules	0.98(0.04–0.22)	< .001
Non-solid nodules	0.88(0.04–0.18)	< .001
Lesion size at imaging, in mm	0.91(0.83–1.00)	.041
Imaging finding		
Spiculation		
No	1(reference)	
Yes	0.54(0.33–0.89)	.015
Pleural retraction		
No	1(reference)	
Yes	1.11(0.67–1.84)	.683
Vascular convergence		
No	1(reference)	
Yes	0.35(0.21–0.60)	< .001
Vacuolar sign		
No	1(reference)	
Yes	0.50(0.29–0.83)	.008
Location		
Left upper lobe	1(reference)	
Left lower lobe	1.84(0.85–4.02)	.124
Right upper lobe	0.85(0.42–1.74)	.653
Right middle lobe	1.07(0.39–2.89)	.902
Right lower lobe	1.45(0.68–3.07)	.336
Subpleural	2.26(0.13–38.12)	.571
Biomarker^a^		
CEA(*n* = 300)		
Negative	1(reference)	
Positive	0.49(0.22–1.09)	.080
NSE(*n* = 271)		
Negative	1(reference)	
Positive	1.30(0.46–3.72)	.618
CYFRA21-1(*n* = 297)		
Negative	1(reference)	
Positive	2.01(1.07–3.76)	.029
SCC(*n* = 300)		
Negative	1(reference)	
Positive	0.78(0.20–3.04)	.719
Variable	Odds Ratio	*P* Value

Data in parentheses are 95% CIs. *CEA* Carcinoma Embryonic Antigen, *NSE* Neuron Specific Enolase, *CYFRA21-1* Cytokeratin 19 fragment, *SCC* Squamous Cell Carcinoma Antigen

^a^The number of patients for whom histopathological data were available for each molecular marker is given

### Multivariable logistic regression analysis and model validation for identifying benign PNs

**Fig. 3:**
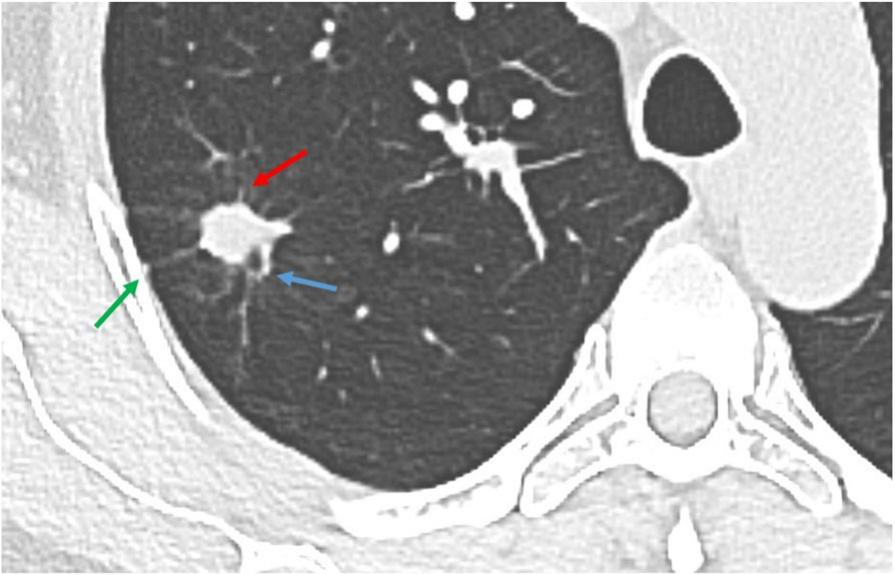
Computed tomography image shows vascular convergence (blue arrows), pleural retraction (green arrows), and spiculation (red arrows). A solid nodule with an average diameter of 1.3 cm in the right upper lobe of a 59-year-old woman’s lung and pathologically confirmed to be benign PN

Based on the results of bivariate logistic regression analysis, a final multivariable logistic regression analysis model was developed to identify benign PNs using the following features: lesion size at imaging; lesion type of part-solid nodules or non-solid nodules; imaging finding of spiculation, vascular convergence, or vacuolar sign; a biomarker of CYFRA21-1 (Table 4). In multivariable analysis, lesion types manifesting as part-solid nodules (OR, 0.14; 95% CI: 0.06, 0.37; *P* < 0.001) and non-solid nodules (OR, 0.05; 95% CI: 0.02, 0.14; *P* < 0.001) remained statistically significant independent factors for benign PNs, which were inversely associated with benign PNs. The area under the curve (AUC) were 0.83 (range, 0.77–0.89) by complete case analysis in the training set. The validation of the predictive model yielded an AUC of 0.88 (95% CI: 0.81, 0.94) (Fig. 3) in the validation set, demonstrating the model's effectiveness in predicting benign PNs.

**Table 4:** Multivariable logistic regression analysis to predict benign nodules in training set

	Multivariable Analysis	
Variable	Odds Ratio	*P* Value
Lesion type		
Part-solid nodules	0.14(0.06–0.37)	< .001
Non-solid nodules	0.05(0.02–0.14)	< .001
Lesion size at imaging, in mm	0.88(0.77–1.00)	.046
Imaging finding		
Spiculation	0.64(0.27–1.52)	.314
Vascular convergence	0.60(0.23–1.59)	.306
Vacuolar sign	1.27(0.56–2.89)	.567
Biomarker^a^		
CYFRA21-1 status (*n* = 297)		
Positive	1.27(0.59–2.75)	.545

Data in parentheses are 95% CIs

^a^The number of patients for whom histopathological data were available for each molecular marker is given

### Analysis of over-diagnosed benign PNs: bivariate and multivariable analyses

**Fig. 4:**
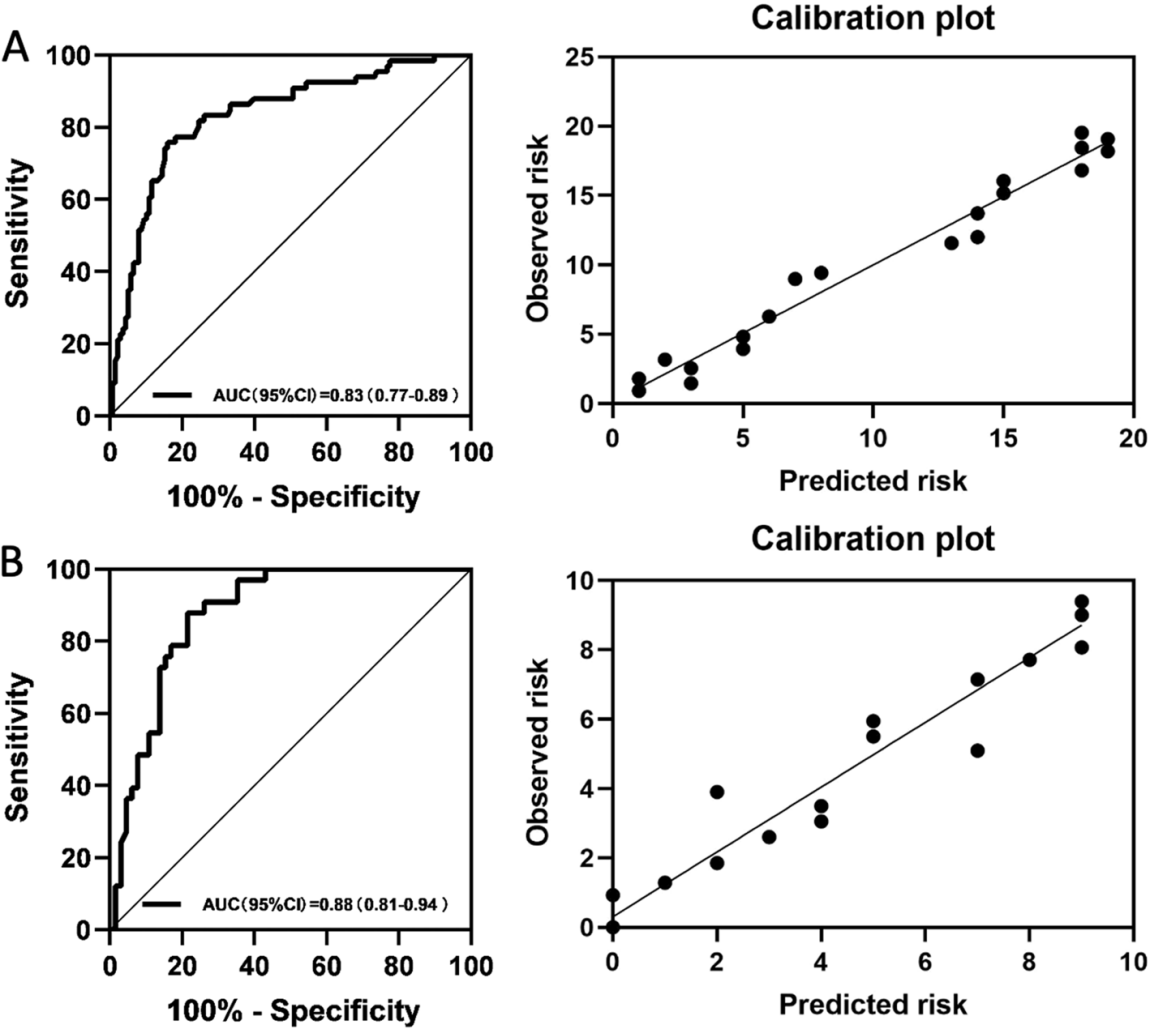
Receiver operating characteristics curves with calibration plots representing the discriminatory ability of the predictive model for benign PNs in (**A**) training (*n* = 285) and (**B**) validation sets (*n* = 122) by using complete case analysis. AUC = area under the receiver operating characteristic curve

**Fig. 5:**
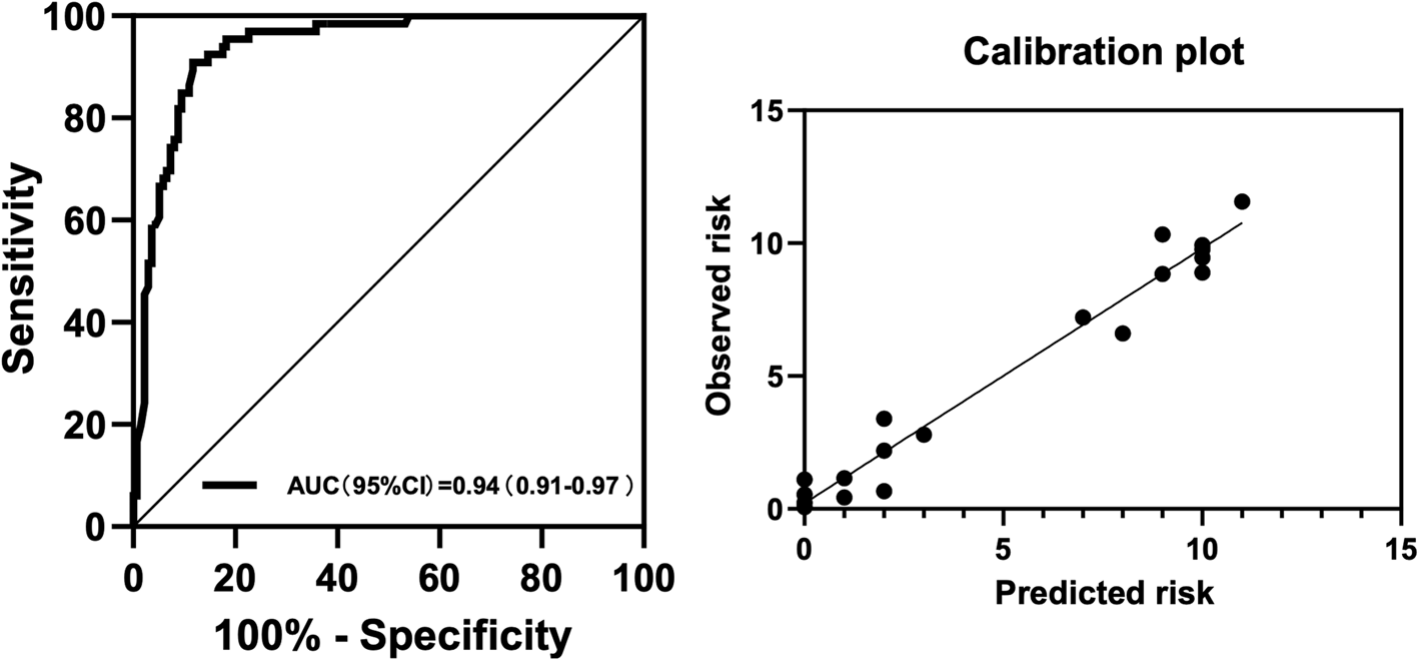
Receiver operating characteristic curves with calibration plots representing the discriminatory power of the over-diagnosed PNs prediction model using complete case analysis in malignant PNs. AUC = area under the receiver operating characteristic curve

Among benign PNs at biopsy, 50% (80 of 160) were diagnosed as malignant PNs by the radiologist on CT scan. Clinical-pathologic and imaging features associated with benign PNs over-diagnosed as malignant PNs are shown in Table 5. At bivariate analysis, part-solid nodules (OR, 4.38; 95% CI: 1.15, 16.73; *P* = 0.031), non-solid nodules (OR, 2.50; 95% CI: 1.07, 5.83; *P* = 0.034), lesion size (OR, 1.42; 95% CI: 1.23, 1.63; *P* < 0.001),spiculation (OR, 16.24; 95% CI: 7.45, 35.41; *P* < 0.001), pleural retraction (OR, 2.76; 95% CI: 1.40, 5.44; *P* = 0.003), vascular convergence (OR, 25.29; 95% CI: 10.45, 61.21; *P* < 0.001), and vacuolar sign (OR, 11.07; 95% CI: 4.97, 24.65; *P* < 0.001)were positively associated with benign PNs which were over-diagnosed. NSE positivity (OR, 0.12; 95% CI: 0.02, 0.69; *P* = 0.018) was inversely associated with benign PNs which were over-diagnosed. On multivariable analysis (Table 5), non-solid nodules (OR, 9.41; 95% CI: 1.93, 45.91; *P* = 0.006), lesion size (OR, 20.42; 95% CI: 2.35, 177.79; *P* = 0.006), pleural retraction (OR, 4.23; 95% CI: 1.07, 16.74; *P* = 0.040), and vascular convergence (OR, 6.64; 95% CI: 1.84, 23.97; *P* = 0.004)were independently associated with benign PNs which were over-diagnosed (Figs. 4 and 5).

**Table 5:** Multivariable logistic regression analysis to identify factors for benign nodules are diagnosed as malignant nodules

	All(*n* = 160)		Bivariate Analysis		Multivariable Analysis	
Variable	Same (*n* = 80)	Higher(*n* = 80)	Odds Ratio^a^	*P* Value	Odds Ratio^a^	*P* Value
Sex						
Male	28(35)	34(42.5)	1 (reference)			
Female	52(65)	46(57.5)	1.37(0.73–2.60)	.331		
Smoking history						
no	60(75)	57(71.3)	1 (reference)			
yes	20(25)	23(28.7)	1.21(0.60–2.44)	.593		
Family history of cancer						
no	13(16.3)	7(8.8)	1 (reference)			
yes	67(83.8)	73(91.3)	0.49(0.19–1.31)	.157		
Lesion type						
Solid nodules	67(83.8)	51(63.8)	1 (reference)			
Part-solid nodules	3(3.8)	19(23.8)	4.38(1.15–16.73)	.031	2.17(0.34–13.74)	.412
Non-solid nodules	10(12.5)	10(12.5)	2.50(1.07–5.83)	.034	9.41(1.93–45.91)	.006
Median lesion size at imaging (mm)	7^b^(6–9)	9†(8–12)	1.42(1.23–1.63)	< .001	20.42(2.35–177.79)	.006
Location						
Left upper lobe	16(20)	12(15)	1 (reference)			
Left lower lobe	14(17.5)	14(17.5)	1.33(0.47–3.82)	.592		
Right upper lobe	21(26.3)	32(40)	2.03(0.80–5.15)	.135		
Right middle lobe	7(8.8)	5(6.3)	0.95(0.24–3.75)	.944		
Right lower lobe	20(25)	17(21.3)	1.13(0.42–3.05)	.804		
Subpleural	2(2.5)	0(0)	0	.999		
Imaging finding						
Spiculation						
No	62(77.5)	14(17.5)	1 (reference)			
yes	18(22.5)	66(82.5)	16.24(7.45–35.41)	< .001	3.24(0.93–11.62)	.064
Pleural retraction						
No	61(76.3)	43(53.8)	1 (reference)			
yes	19(23.8)	37(46.3)	2.76(1.40–5.44)	.003	4.23(1.07–16.74)	.040
Vascular convergence						
No	59(73.8)	8(10)	1 (reference)			
yes	21(26.3)	72(90)	25.29(10.45–61.21)	< .001	6.64(1.84–23.97)	.004
Vacuolar sign						
No	70(87.5)	31(38.8)	1 (reference)			
yes	10(12.5)	49(61.3)	11.07(4.97–24.65)	< .001	1.59 (0.39–6.47)	.520
Biomarker positive^c^						
CEA status						
Negative	46(79.3)	50(84.7)	1 (reference)			
Positive	12(20.7)	9(15.3)	0.69(0.27–1.79)	.445		
NSE status						
Negative	42(79.2)	50(98)	1 (reference)			
Positive	11(20.8)	1(2)	0.12(0.02–0.69)	.018	0.19(0.02–1.96)	.162
CYFRA21-1 status						
Negative	39(72.2)	34(57.6)	1 (reference)			
Positive	15(27.8)	25(42.4)	1.87(0.86–4.06)	.116		
SCC status						
Negative	57(98.3)	56(94.9)	1 (reference)			
Positive	1(1.7)	3(5.1)	3.05(0.31–30.25)	.340		

Unless otherwise noted, variables are expressed as numbers of patients with percentages in parentheses, or as medians, with interquartile ranges in parentheses. *CEA* Carcinoma Embryonic Antigen, *NSE* Neuron Specific Enolase, *CYFRA21-1* Cytokeratin 19 fragment, *SCC* Squamous Cell Carcinoma Antigen

^a^Data in parentheses are 95% CIs

^b^Data are median millimeters; data in parentheses are interquartile range

^c^The number of patients for whom histopathological data were available for each molecular marker is given

## Discussion

In this study, we identified specific preoperative features for evaluating benign pulmonary nodules (PNs) that were confirmed by surgery or biopsy. Lesions that were neither part-solid nor non-solid nodules, large lesion size, and the absence of spiculation and vascular convergence at CT scan were significantly associated with benign PNs. These features were validated as successful predictors of benign PNs in the validation set. The overdiagnosis rate of benign PNs at imaging assessment as malignant PNs was 50.0%. Non-solid nodules, lesion size, spiculation, pleural retraction, and vascular convergence were positively associated with overdiagnosis of benign PNs as malignant PNs at surgery or biopsy.

Although the benefits of lung cancer screening and diagnosis of early pulmonary cancer have largely been demonstrated [19], we still consider the risk of overdiagnosis based on highly benign nodule surgery rates. Some indolent tumors have no impact on the patients’ lives even if left untreated [20] and we focus on anxiety and unnecessary invasive treatment brought to patients. Invasive treatment of benign PNs might not increase the prognosis of treatment and can cause complications in patients undergoing of biopsy or surgery. Transthoracic core needle aspiration biopsy and fine-needle aspiration (FNA) are performed under CT guidance to obtain tissue. Core biopsies are superior to FNA because of their higher yield, but more importantly, biopsies allow the assessment of tissue structure and provide sufficient material for immunohistochemical and genetic analysis. However, complications can occur in transthoracic core needle aspiration biopsy despite all precautions taken. Complications of transthoracic core needle aspiration biopsy include pneumothorax, hemothorax, hemoptysis, infection, tumor spreading, and air embolism, with the most common complication as pneumothorax. According to a population-level retrospective cohort analysis,16,971 patients underwent transthoracic core needle aspiration biopsy, and 25.8% experienced a complication within 3 days of the procedure (pneumothorax 23.3%, hemorrhage 3.6%, and air embolism 0.02%) [21]. Several lately studies [22, 23] have been evaluating the complications and risk–benefit of benign PNs which were treated with VATS. It is essential to discriminate benign PNs preoperatively because the complications of VATS might cause some irreversible injury in clinical practice. In recent studies, VATS has been shown to cause complications in 33.9% of patients at 90 days post-operatively, which has no significant differences with thoracotomy [23]. In our study, the patients were divided into a training set and a validation set, and the imaging features and biomarkers for differentiating benign and malignant PNs were obtained. Multivariate analysis showed that ground glass nodule or non-solid nodule, no spiculation, no vacuolar sign, vascular convergence, large lesion size and positive CYFRA21-1 were still independent factors for the differential diagnosis of benign nodules. Then, our model identified the final type of PNs based on preoperative results with an area under the receiver operating characteristic curve of 0.88 in the validation set. Similarly, previous research has also proven that type of PNs and image findings of spiculation, vascular convergence and vacuolar sign are independent risk factors for pulmonary cancer [24–26]. Other studies have similarly shown the importance of the type of PNs and image findings of spiculation and vascular convergence in the diagnosis of benign and malignant PNs [27–29]. Several studies [30, 31] have been proven the value of combining with CEA, CYFRA21-1 and NSE, but no significant association was found in our study.

In our study, 50% of benign PNs were over-diagnosed as malignant at the time of imaging assessment which was not negligibly high; if the PN is found to be non-solid with spiculation, pleural retraction, vascular convergence, and larger lesion size at the time of CT scan, the possibility of overdiagnosis of PNs can be reconsidered. At the same time, attention should be taken not to excessively increase the proportion of PN size in the judgment of benign and malignant PNs. Although we anticipated that the patient's age and smoking history, the location of the PN, and biomarkers might influence the judgment of overdiagnosis or not, this hypothesis was not supported after multivariate analysis. This might be because the number of our study population was not large enough and the biomarkers were not highly sensitive to overall malignant nodules but were sensitive to specific subtypes. Biomarkers, as a means of cancer screening, take advantage of the characteristics of minimally invasive. However, conventional tumor markers (CEA, NSE, CYFRA21-1, and SCC) appear to be sensitive only to certain types of tumors or require a large enough tumor volume to produce, so they are not sensitive in PNs ranging from 5 to 15 mm. However, a study [32] has found that the expression of specific biomarkers such as plasma proteins LG3BP and C163A, combined with age, smoking status, nodule diameter, shape, and location, has a good ability to distinguish benign and malignant PNs. The popularization of special biomarkers may increase the accuracy of differentiating benign and malignant PNs, but it also puts forward higher requirements for the ability to discriminate. Overall, considering that it is unacceptable to miss the diagnosis of malignant PNs, clinicians and radiologists are still debating the appropriate treatment strategy for 5 mm to 15 mm PNs.

We believe our model can be used before surgery to help clinicians decisively select lesions that are likely to be benign. In our study, we focused on NCCT examination features. PNs type and size are important influences on the classification of PNs, and nodules' type can be depicted by dual-energy CT [33]. A study found that adenocarcinoma in situ (AIS) and minimally invasive adenocarcinoma (MIA) patients had a 100% 5-year recurrence-free rate after resection of PNs, suggesting that it is important to distinguish AIS and MIA from other malignant nodules [34]. We believe that there is potential for better prediction of PNs to distinguish AIS and MIA and protect patients from non-essential invasive treatment in the future through increased use of dual-energy CT features.

Our study had several limitations. First, it was conducted at a single institution, and we did not perform external validation from an external institution. The sample inevitably increased the proportion of oncology patients, due to our institution being an oncology specialized hospital. Second, the rate of overdiagnosis in this study may be higher than that of its providers due to the high cost of missed diagnoses and physicians' hypersensitivity to high-risk lesions. Third, the different CT machine models and scanning parameters used in this study may lead to the lack of standardization of image details. Last, due to the lack of unified standards for patient examination, the data such as biomarkers are missing, which ultimately leads to an unsatisfactory sample size.

## Conclusion

In conclusion, a large number of patients with benign PNs in the clinic were over-diagnosed as malignant nodules in imaging, and unnecessary surgery or core biopsy intervention was performed. This preoperative model and the factors that led to the overdiagnosis of benign PNs may help clinicians reduce unnecessary surgery and help imaging physicians make more accurate diagnosis.

## Data Availability

The datasets used and analyzed during the current study are available from the corresponding author on reasonable request.

## Abbreviations

AIS: Adenocarcinoma in situ
AUC: Area under the curve
CT: Computed tomography
FNA: Fine-needle aspiration
LDCT: Low-dose CT
MIA: Minimally invasive adenocarcinoma
NCCT: Non-contrast CT
PN: Pulmonary nodule
ROC: Receiver operating characteristic curve
