# Publisher Correction: Factors associated with overdiagnosis of benign pulmonary nodules as malignancy: a retrospective cohort study

**DOI:** 10.1186/s12890-023-02801-0

**Published:** 2023-12-14

**Authors:** Xirui Duan, Zhiqiang Ouyang, Shasha Bao, Lu Yang, Ailin Deng, Guangrong Zheng, Yu Zhu, Guochen Li, Jixiang Chu, Chengde Liao

**Affiliations:** 1 Department of Radiology, Yan?an Hospital of Kunming City (Yan?an Hospital Affiliated to Kunming Medical University; Yunnan Cardiovascular Hospital), Kunming, China; 2 Department of Radiology, Yunnan Cancer Hospital/Center, Third Affiliated Hospital of Kunming Medical University, Kunming, China

**BMC Pulmonary Medicine (2023) 23:454**

10.1186/s12890-023-02727-7

Following publication of the original article, an error in the author list was brought to the attention of the journal: the first two authors had not been marked as co-first authors. The published article has since been corrected. The publisher thanks you for reading this erratum and apologizes for any inconvenience caused.

